# Comparison of single-spin to double-spin platelet-rich plasma centrifugation methods in the treatment of androgenic alopecia: a systematic review and meta-analysis of randomized controlled trials

**DOI:** 10.3389/fmed.2025.1631087

**Published:** 2025-07-21

**Authors:** Laura Ghanem, Najwaa Kirmani, María Paula Palacios-Ortiz, Martin Cevallos-Cueva, Daniela Lucía Mendoza-Millán, Gabriel Victor Almeida Nascimento, Virginia Velasco-Tamariz

**Affiliations:** ^1^Faculty of Medical Sciences, Lebanese University, Beirut, Lebanon; ^2^Dow Medical College, Karachi, Pakistan; ^3^School of Medicine, Universidad San Francisco de Quito, Quito, Ecuador; ^4^School of Medicine, Universidad Central del Ecuador, Quito, Ecuador; ^5^Department of Dermatology, Harvard Medical School, Boston, MA, United States; ^6^Department of Dermatology, Beth Israel Deaconess Medical Center, Boston, MA, United States; ^7^Faculty of Medicine, Universidade Ceuma Campus Imperatriz, Imperatriz, Maranhão, Brazil; ^8^Dermatology Department, Hospital Universitario 12 de Octubre, I+12 Research Institute, Universidad Complutense de Madrid, Madrid, Spain

**Keywords:** androgenic alopecia, platelet-rich plasma, centrifugation, single-spin, double-spin

## Abstract

**Background:**

Androgenic alopecia (AGA) is the most common hair loss disease, caused by a combination of genetic factors and hormonal imbalances. Platelet-rich plasma (PRP) therapy has gained significant recognition in treating AGA, but optimal treatment protocol regarding centrifugation techniques has yet to reach a standardized consensus. We aim to conduct a systematic review and meta-analysis of randomized controlled trials (RCTs) to compare the efficacy of single-spin versus double-spin centrifugation protocols for PRP in patients with AGA.

**Methods:**

We searched PubMed, Embase, and Cochrane for RCTs comparing single-spin to double-spin PRP in AGA patients. The primary endpoint was the final platelets count after centrifugation. Secondary endpoints included improvement in hair density, as well as terminal and vellus hair rate. Statistical analysis was performed using RStudio software. Heterogeneity was assessed with *I*^2^ statistics.

**Results:**

We included 90 participants with AGA from three RCTs. The pooled mean difference (MD) for platelets concentration was 66.14 in single-spin compared to double-spin PRP (95% CI: −372.96 to 505.25; *p* = 0.77; *I*^2^ = 83.5%). The MD for change in hair density was 4.10% in single versus double-spin (95% CI: −4.74 to 12.93; *p* = 0.36; *I*^2^ = 0%). Moreover, the MD for terminal and vellus hair after single-spin versus double-spin PRP was 1.16 (95% CI: −3.34 to 5.66; *p* = 0.61; *I*^2^ = 0%), and −3.59 (95% CI: −12.49 to 5.30; *p* = 0.45; *I*^2^ = 4.8%), respectively.

**Conclusion:**

Our study suggests that single-spin PRP centrifugation is better in treating AGA patients compared to double-spin PRP.

**Systematic review registration:**

https://www.crd.york.ac.uk/PROSPERO/view/CRD42025643296, identifier CRD42025643296.

## 1 Introduction

Androgenic alopecia (AGA), the most common type of hair loss, is driven by a combination of genetic factors and hormonal imbalances (1). Increased levels of dihydrotestosterone (DHT), along with upregulated androgen receptors and caspases, contribute to cellular apoptosis and progressive hair thinning (1, 2). As DHT levels rise, the anagen phase of hair growth becomes shorter, while hair follicles in androgen-sensitive areas undergo miniaturization. Consequently, the hair becomes thinner, loses its pigment, and eventually transforms into vellus hair (1, 3).

Platelet-rich plasma (PRP) therapy has gained significant recognition in many fields, particularly in the treatment of AGA. Platelets are known to release multiple growth factors (GFs), such as platelet-derived growth factor (PDGF), vascular endothelial growth factor (VEGF), and transforming growth factor beta (TGF-β), which facilitate cell proliferation, collagen synthesis, and angiogenesis (4, 5). It is suggested that PRP promotes the hair growth cycle, leading to increased hair density and shaft diameter, specifically promoting the growth of terminal hair, which is thick and pigmented (4, 6).

One of the key variables in PRP preparation is the centrifugation protocol. PRP can be prepared using either a single-spin or double-spin method, each yielding products with different cellular compositions. Single-spin techniques are simpler and quicker, typically resulting in higher platelet recovery but may retain more leukocytes. In contrast, double-spin protocols are designed to concentrate platelets while reducing leukocyte content, making them the preferred choice when leukocyte-poor PRP is desired – such as in aesthetic and dermatologic applications to minimize pro-inflammatory effects (7, 8).

Beyond AGA, the effects of centrifugation technique on PRP quality and clinical outcomes have been studied in various fields including orthopedics, dentistry, and sports medicine. These studies demonstrate that centrifugation strategy can significantly influence platelet concentration, GFs levels, and therapeutic efficacy depending on the clinical context (9–11). Despite the growing use of PRP in AGA, the efficacy and optimal treatment protocol regarding centrifugation techniques have yet to reach a standardized consensus, largely due to the insufficiency of comparative data on different centrifugation protocols (8, 12). We hypothesized that the choice of centrifugation method – single-spin versus double-spin – affects both platelet concentration and clinical outcomes in patients with AGA, with potential implications for therapeutic efficacy.

To our knowledge, this is the first systematic review and meta-analysis focusing specifically on randomized controlled trials (RCTs) comparing the efficacy of single-spin versus double-spin centrifugation protocols for PRP in patients with AGA. By providing a focused, evidence-based comparison, our study addresses a significant gap in the literature and contributes to the ongoing effort to standardize PRP protocols in hair restoration.

## 2 Methods

This Meta-Analysis and Systematic Review was registered in the International Prospective Register of Systematic Reviews (PROSPERO) under registration number (CRD42025643296), performed under the Cochrane Handbook for Systematic Reviews of Interventions, and reported adhering to the Preferred Reporting Items for Systematic Reviews and Meta-Analyses (PRISMA) statement guidelines (13).

### 2.1 Eligibility criteria

Studies that met the following eligibility criteria were included: (1) RCTs; (2) comparing single-spin to double-spin centrifugation methods for PRP; (3) reporting the results in patients with AGA; and (4) reporting at least one clinical outcome of interest. No restrictions were applied to follow-up time or language. We excluded studies with: (1) overlapping patient populations; (2) observational design or of within-person RCT design; (3) had overlapping patient populations; (4) had no control group; (4) were performed in animals; (5) did not include patients with alopecia; and (7) with missing data on the interventional therapy used.

### 2.2 Search strategy and data extraction

We systematically searched PubMed, Embase, and Cochrane Central Register of Controlled Trials from inception to February 2025, using the following search terms: “alopecia,” “hair,” “baldness,” “platelet-rich plasma,” “platelet-enriched plasma,” “thrombocyte-rich plasma,” “autologous conditioned plasma,” “PRP,” “spin,” “spinning,” “centrifugation,” “centrifugal,” “centrifuge,” “separation,” “single-spin,” “one-spin,” “double-spin,” and “two-spin.” Boolean operators like “OR” and “AND” were used to optimize the search results. The complete search strategy can be found in the Supplementary Appendix 1. The references from all included studies, previous systematic reviews, and meta-analyses were also searched manually for any additional studies. Two authors (L.G. and N.K.) independently extracted the data following predefined search criteria and quality assessment. Disagreements were resolved by consensus among the two authors (L.G. and N.K.).

### 2.3 Endpoints

The primary endpoint was final platelet counts after centrifugation. Secondary endpoints were the percentage of change in hair density, the rate of final terminal hair, and the rate of vellus hair. The methods used for hair evaluation included standardized digital photography and analysis tools such as the Canon EOS 50D SLR with dermatoscope and NEO Image Analysis Software (14), and the FotoFinder^®^ medicam 1000s, trichoscopy, and TrichoScan (15). In the study by Moftah et al. (16), we excluded group IV (single-spin) because it used a digital centrifuge, unlike the other three groups in that study, which used an electronic centrifuge. We made this exclusion to minimize heterogeneity due to differences in centrifuge settings and potential confounding factors. Moreover, we kept group II (double-spin) for comparison with group I (single-spin), as its second spin at 2,000 rpm was closer to the centrifugation speed used in the other included RCTs (16).

### 2.4 Statistical analysis

Mean difference (MD) and SD with 95% confidence intervals were used to compare the efficacy of single-spin PRP with double-spin PRP in AGA. Heterogeneity was assessed with *I*^2^ statistics and Cochran *Q* test; *p* values < 0.10 and *I*^2^ > 25% were considered significant for heterogeneity. For continuous outcomes, the inverse-variance method (IV) with REML was applied (17). A random-effects model was used for forest plots that included three studies, while a fixed-effects model was applied for forest plots that included two studies (18). We used RStudio software (version 2024.09.1+394) for statistical analysis.

### 2.5 Quality assessment

Cochrane Collaboration’s tool for assessing risk of bias in randomized trials (RoB 2) was used to assess the quality of individual RCTs (19). Two independent authors completed the risk of bias assessment (M.C.-C. and G.A.). Disagreements were resolved through a consensus after discussing reasons for the discrepancy.

## 3 Results

### 3.1 Study selection and baseline characteristics

**FIGURE 1:**
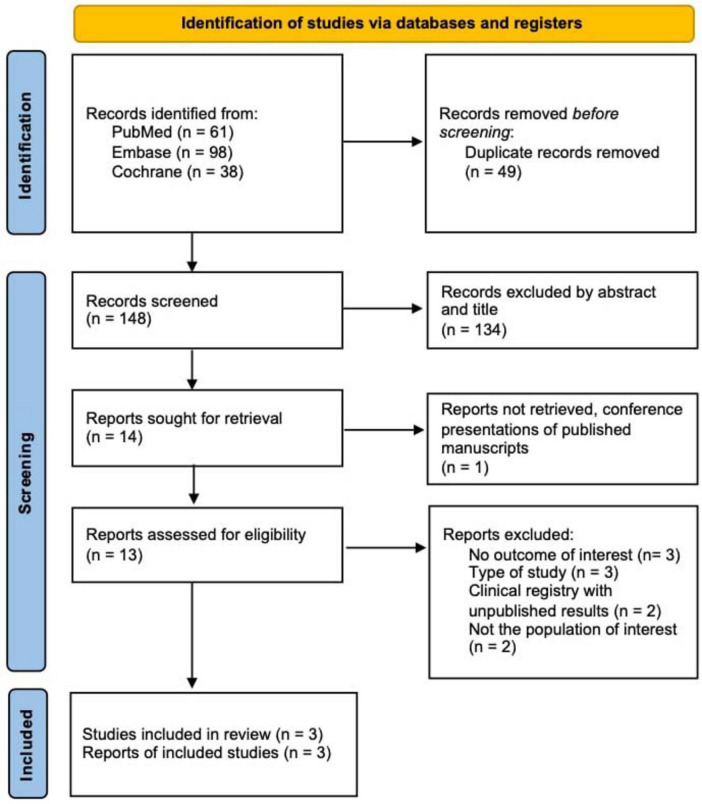
PRISMA flow diagram of study screening and selection.

The initial search yielded 197 results. After removal of duplicate records and ineligible studies, 13 remained and were fully reviewed based on inclusion criteria. Of these, a total of three RCTs were included in this meta-analysis, comprising a total of 90 participants diagnosed with AGA (Figure 1). Among the participants, 70 were male (77.8%). The baseline characteristics of the included studies, including PRP centrifugation parameters, alopecia classification systems, and follow-up durations, are summarized in Table 1. The follow-up periods ranged from 6 weeks to 12 months, providing a comprehensive view of short- and long-term outcomes.

**TABLE 1:** Baseline characteristics of included studies.

Study	Volume of PRP drawn per patient (ml)	Platelet activator	Single-spin (speed/time)	Double-spin (speed/time)	Follow-up (months)	No. of patients, *n*	No. of male patients, *n* (%)	Age (years), range, SD	Platelet count (thousand/μ l), mean	Alopecia classification	PRP regimen
Ince et al. (14)	4–5	Calcium chloride	3,000 rpm for 15 min	1st spin at 3,000 rpm for 15 min 2nd spin at 2,000 rpm for 5 min	12	40	40 (100)	29.95	272.5	Type 2–4 (Hamilton-Norwood)	1, 2, and 6 months after 1st injection
Moftah et al. (16)	10	None	900 or 3,000 rpm for 10 min	1st spin at 1,500 rpm for 10 min 2nd spin at 2,000 or 3,000 rpm for 5 min	4	40	0 (0)	25.75 ± 4.61	284.35	Grade I and II (Ludwig)	1 and 2 months after 1st injection
Legiawati et al. (15)	13.5	Calcium chloride or calcium gluconate	3,000 rpm for 15 min	1st spin at 1,500 rpm for 6 min 2nd spin at 2,500 rpm for 5 min	1.5	30	30 (100)	34.57 ± 7.53	307.66	Stages III and VI (Hamilton–Norwood)	2 and 4 weeks after 1st injection

rpm, revolutions per minute; PRP, platelet-rich plasma; SD, standard deviation. Definitions of alopecia classification type varied between studies. The study by Moftah et al. (16), which included only female patients, used the Ludwig scale ranging from I to III. Grade I: perceptible thinning of the hair on the crown, limited in the front by a line situated 1–3 cm behind the frontal hairline. Grade II: pronounced rarefaction of the hair on the crown within the area seen in grade I. Grade III: full baldness (total denudation) within the area seen in grades I and II (33). On the other hand, the two other studies (14, 15) that included only male patients, used the Hamilton–Norwood scale ranging from I to VII. Stage II: the frontotemporal recession of the hairline does not extend further than 2 cm anterior to the mid-coronal line. Stage III: the frontotemporal recession of the hairline extends further than type II and may reach the midcoronal line. Stage IV: the frontotemporal recession of the hairline has receded beyond the mid-coronal line. Stage VI: the frontotemporal and vertex regions of the alopecia have become confluent, and the area of alopecia has increased laterally and posteriorly (34–36).

### 3.2 Pooled analysis of all studies

#### 3.2.1 Platelet concentration

**FIGURE 2:**
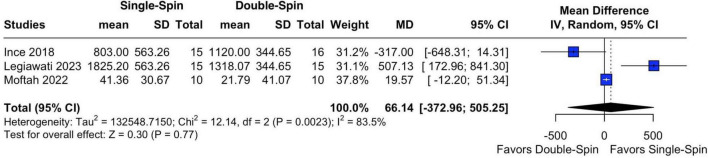
Final platelets count after centrifugation (all values expressed in ×10^9^/L. SD, standard deviation; MD, mean difference; CI, confidence interval; IV, inverse variance.

The three studies provided data on final platelet counts after centrifugation (14–16). A high degree of heterogeneity (*I*^2^ = 83.5%) was observed, likely attributable to differences in centrifuge settings and protocols across studies. The pooled MD for platelets concentration in single-spin versus double-spin centrifugation method was 66.14 (95% CI: −372.96 to 505.25; *p* = 0.77; Figure 2) (*all values expressed in* × *10^9^/L)*, indicating no significant differences between single-spin and double-spin methods.

#### 3.2.2 Hair density improvement

**FIGURE 3:**
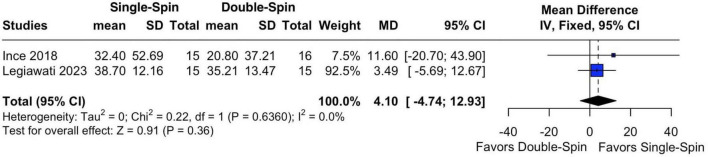
Percentage of change in hair density after PRP. SD, standard deviation; MD, mean difference; CI, confidence interval; IV, inverse variance.

The pooled analysis of hair density improvement included two studies (14, 15). The pooled MD for percentage change in hair density in single versus double-spin PRP was 4.10 (95% CI: −4.74 to 12.93; *p* = 0.36; Figure 3), with no significant heterogeneity (*I*^2^ = 0%). These results suggest no statistically significant difference in hair density improvement between single-spin and double-spin PRP centrifugation methods.

#### 3.2.3 Terminal hair count and vellus hair count

**FIGURE 4:**
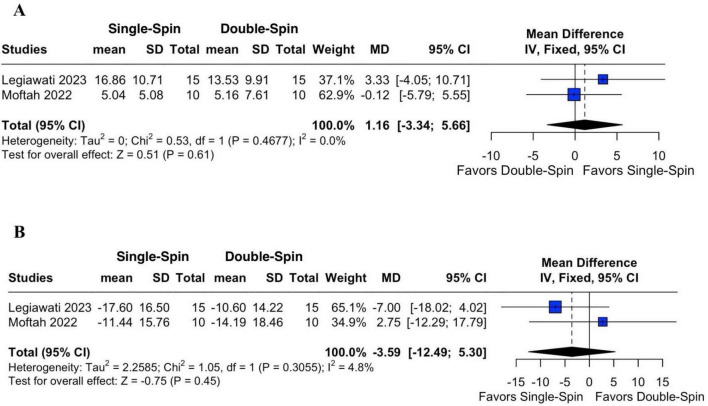
Percentage of change in terminal **(A)** and vellus hair count **(B)** after PRP. SD, standard deviation; MD, mean difference; CI, confidence interval; IV, inverse variance.

For terminal hair count differences, two studies were analyzed (15, 16). The pooled MD for the percentage change in terminal hair count in single-spin PRP, compared to double-spin PRP was 1.16 (95% CI: −3.34 to 5.66; *p* = 0.61; Figure 4A), with no heterogeneity detected (*I*^2^ = 0%).

For vellus hair count also reported by two studies, the pooled MD was −3.59 (95% CI: −12.49 to 5.30; *p* = 0.45; Figure 4B), with negligible heterogeneity (*I*^2^ = 4.8%) in single-spin versus double-spin PRP (15, 16). These findings suggest that neither centrifugation method had a statistically significant advantage in improving terminal or vellus hair counts.

### 3.3 Quality assessment

**FIGURE 5:**
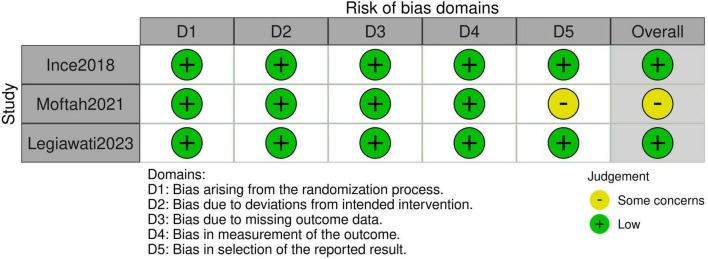
Risk of bias assessment per RoB 2.

The risk of bias assessment, performed using the RoB 2 tool, is summarized in Figure 5. Two studies were rated as having a low risk of bias across all domains (14, 15). Moftah et al. (16) had some concerns regarding the “bias in the selection of the reported result” domain, as the trial was not registered on an official registry prior to initiation, despite obtaining ethical approval. However, the study was otherwise assessed as having a low risk of bias (16).

## 4 Discussion

In this meta-analysis of three studies, we compared single-spin to double-spin PRP centrifugation methods in patients with AGA. Key findings include: (i) higher platelets count with single-spin PRP; (ii) higher hair density improvement in the single-spin group; (iii) slightly higher terminal hair count with one-spin centrifugation; and (iv) a lower vellus hair count with single-spin PRP. Our findings favor single-spin PRP over double-spin PRP showing a consistent trend of increased hair density, terminal hair growth and reduction in vellus hair, however the results were not statistically significant.

Platelet-rich plasma plays a crucial role in hair restoration by leveraging the regenerative properties of platelets and its rich concentration of GFs and cytokines. These factors include PDGF, VEGF, TGF-β, fibroblast growth factor (FGF), epidermal growth factor (EGF), and insulin-like growth factor 1 (IGF-1). Upon activation, platelets release bioactive proteins that target and bind to receptors on stem cells, triggering intracellular pathways in the hair follicle bulge. This enhances GF activity in dermal papilla cells, prolongs the growth phase, known as the anagen phase, stimulates bulge cell differentiation, and activates the Wingless/Integrated and β-catenin (Wnt/β-catenin) and extracellular signal-regulated kinase/protein kinase B/Akt (ERK/PKB/Akt) signaling pathways, which support follicle development and prevent apoptosis, through B-cell lymphoma 2 (Bcl-2) expression. PRP also enhances anagen-associated angiogenesis, driven by VEGF secreted by keratinocytes and dermal papilla fibroblasts (20, 21).

A within-person RCT, where one half of the scalp received single-spin PRP and the other half received double-spin PRP, demonstrated superior hair regrowth with the double-spin technique, as confirmed by trichoscopic assessment (22). However, we excluded this study from our meta-analysis due to significant methodological concerns highlighted in a systematic review by Leducq et al. (23). Notably, the risk of carry-across effects, where treatment on one site may inadvertently influence adjacent or contralateral areas, posed a major confounding factor. To ensure methodological rigor and data reliability, we included only traditional parallel-group RCTs in our analysis, allowing for independent comparisons of interventions.

The Indian Association of Dermatologists, Venereologists, and Leprologists (IADVL) developed standardized guidelines from 45 studies. The consensus supports the double-spin manual method, recommending centrifugation parameters of 100–300 *g* for 5–10 min in the first spin and 400–700 *g* for 10–17 min in the second. However, the review highlights inconsistencies in existing methodologies and the need for standardization to ensure reproducibility and efficacy in PRP treatments (8).

Despite the widely reported effectiveness of PRP therapy for AGA, significant clinical benefits have not been well established in the current literature, highlighting inconsistencies in treatment outcomes. For instance, a study was conducted on 50 patients [38 males with grade II–V (Norwood–Hamilton) and 12 females with grade II–III (Ludwig)], who underwent PRP treatment with a double-spin centrifugation method injected. Evaluation through photography follow-ups and patient satisfaction surveys revealed that 19 patients were unsatisfied and only 8 found the treatment satisfactory (24). Similarly, a trial comparing double-spin PRP to placebo on 26 females with Ludwig II AGA, failed to demonstrate PRP’s efficacy, with therapeutic benefits reported in patient surveys but not supported by objective hair count data or the hair mass index (HMI) (25). Additionally, a pilot study involving 19 males with AGA grade IV–VI (Norwood classification), used a two-step centrifugation process that increased platelet concentration by threefold. There was no increase in hair count, and conversion of vellus hair to terminal hair was not observed (26).

Notably, these three studies comparing double-spin PRP to placebo showed no benefit, suggesting its inefficacy may stem from centrifugation methodology—especially as our meta-analysis favored single-spin PRP. However, given the lack of statistical significance, further studies directly comparing both methods are needed. Establishing a standardized PRP protocol is crucial to optimizing outcomes and ensuring patient-centered care.

The activation state of PRP, whether in its natural or activated form, significantly influences its therapeutic efficacy. Activated PRP (A-PRP), induced by thrombin or calcium chloride, provides a controlled and sustained release of GFs, optimizing regenerative outcomes. By contrast, exogenously activated PRP (AA-PRP) results in a rapid but short-lived release, potentially limiting long-term effectiveness. This study further emphasized the superior outcomes of A-PRP in improving hair density and follicular activity, mediated by pathways such as Bcl-2, Akt signaling, and FGF-7 activation (27). Future trials comparing single-spin and double-spin PRP should prioritize A-PRP to standardize protocols, as it has demonstrated superior efficacy. This approach will help refine treatment guidelines and optimize clinical outcomes.

Platelet-rich plasma therapy has demonstrated efficacy not only in AGA but also in challenging hair conditions such as cicatricial centrifugal alopecia, including central centrifugal cicatricial alopecia (CCCA), and chronic telogen effluvium (CTE). Case studies on CCCA revealed temporary improvements in follicular density during monthly PRP sessions. However, sustained efficacy remains uncertain, underscoring the need for shorter maintenance intervals (28). In cases of CTE, PRP therapy improved hair density, thickness, and trichoscopy findings, regardless of the type of PRP tube used. Notably, no significant differences were observed between single-spin and double-spin methods (29). Given these findings, further RCTs are essential to compare single-spin versus double-spin PRP centrifugation techniques. Establishing optimal protocols for PRP regimens across these populations would further enhance clinical outcomes.

Combining PRP therapy with standard alopecia treatments – such as minoxidil, oral medications, microneedling, and non-cross-linked hyaluronic acid – has demonstrated synergistic effects on hair regrowth and scalp health. Studies indicate that PRP enhances follicular stimulation, while microneedling improves drug absorption, and hyaluronic acid provides hydration, collectively increasing hair density and thickness (30, 31). These findings underscore that PRP not only enhances the effectiveness of traditional treatments but also offers a safe, minimally invasive solution with minimal side effects. However, the development of a standardized protocol remains critical to optimize these results and ensure consistent therapeutic outcomes for patients. Therefore, we recommend further trials comparing combined PRP with standard treatments, considering centrifugation protocols to optimize patient outcomes.

This study has several limitations. Firstly, the sample size was relatively small, resulting in statistically non-significant findings, thereby affecting the power and robustness of our conclusions. Web of Science and Scopus were not included in the search, which could represent a minor limitation in study inclusion. Additionally, only three studies met our inclusion criteria, with a single common outcome reported across all three RCTs. Forest plots were generated using data from only two studies due to the limited availability of outcomes. Moreover, variability in centrifugation speed and duration, PRP regimen, and follow-up periods across studies may have introduced confounding factors. However, we sought to mitigate this by including only RCTs, ensuring that both comparison groups within each study received the same PRP regimen and were followed up for the same duration; and we excluded within-person RCTs to enhance comparability. Another limitation is the absence of reported measures of dispersion for platelet count outcomes in the study by Ince et al. (14). To address this, we imputed the standard deviation using data from Legiawati et al., following Cochrane’s guidelines (15, 32). Furthermore, we did not evaluate other important factors such as PRP activation methods or the combination of PRP with other treatment modalities, which are increasingly used in clinical practice. These aspects were beyond the scope of the current review and should be addressed in future research.

## 5 Conclusion

In conclusion, this systematic review and meta-analysis based on available evidence suggest that the single-spin centrifugation method for PRP has better outcomes than double-spin PRP increasing hair density, terminal hair, and platelets while decreasing thin unpigmented hair, known as vellus hair.

## Data Availability

The original contributions presented in this study are included in this article/Supplementary material, further inquiries can be directed to the corresponding author.
