# Supplementary material for: Comparison of single-spin to double-spin platelet-rich plasma centrifugation methods in the treatment of androgenic alopecia: a systematic review and meta-analysis of randomized controlled trials
Source: Front Med (Lausanne). 2025 Jul 21;12:1631087. doi: 10.3389/fmed.2025.1631087 (PMC12318733; doi:10.3389/fmed.2025.1631087)
Supplement: Supplementary file 1 [file Supplementary_file_1.pdf]

# Comparison of single-spin to double-spin platelet-rich plasma (PRP) centrifugation methods in the treatment of androgenic alopecia: A systematic review and meta-analysis of randomized controlled trials

- **Search Strategy for PubMed**

(alop*cia OR hair OR baldness OR "Alopecia"[Mesh]) AND (“platelet-rich plasma” OR PRP OR “platelet-enriched plasma” OR “thrombocyte-rich plasma” OR “autologous conditioned plasma” OR "Platelet-Rich Plasma"[Mesh]) AND (spin OR spinning OR centrifugation OR centrifugal OR centrifuge OR separation OR “single-spin” OR “one-spin” OR “double-spin” OR “two-spin”)

- **Search Strategy for Embase**

('alop*cia' OR 'hair' OR 'baldness' OR 'alopecia'/exp) AND ('platelet-rich plasma' OR 'prp' OR 'platelet-enriched plasma' OR 'thrombocyte-rich plasma' OR 'autologous conditioned plasma' OR 'thrombocyte rich plasma'/exp) AND ('spin' OR 'spinning' OR 'centrifugation' OR 'centrifugal' OR 'centrifuge' OR 'separation' OR 'single-spin' OR 'one-spin' OR 'double-spin' OR 'two-spin')

- **Search Strategy for Cochrane**

(alop*cia OR hair OR baldness) AND (“platelet-rich plasma” OR PRP OR “platelet-enriched plasma” OR “thrombocyte-rich plasma” OR “autologous conditioned plasma”) AND (spin OR spinning OR centrifugation OR centrifugal OR centrifuge OR separation OR “single-spin” OR “one-spin” OR “double-spin” OR “two-spin”)
